# The Effect of Phylogeny, Environment and Morphology on Communities of a Lianescent Clade (Bignonieae-Bignoniaceae) in Neotropical Biomes

**DOI:** 10.1371/journal.pone.0090177

**Published:** 2014-03-03

**Authors:** Suzana Alcantara, Richard H. Ree, Fernando R. Martins, Lúcia G. Lohmann

**Affiliations:** 1 Departamento de Botânica, Instituto de Biociências, Universidade de São Paulo, São Paulo, SP, Brazil; 2 Department of Botany, Field Museum of Natural History, Chicago, Illinois, United States of America; 3 Departamento de Biologia Vegetal, Instituto de Biologia, Universidade Estadual de Campinas – UNICAMP, Campinas, SP, Brazil; Central China Normal University, China

## Abstract

The influence of ecological traits to the distribution and abundance of species is a prevalent issue in biodiversity science. Most studies of plant community assembly have focused on traits related to abiotic aspects or direct interactions among plants, with less attention paid to ignore indirect interactions, as those mediated by pollinators. Here, we assessed the influence of phylogeny, habitat, and floral morphology on ecological community structure in a clade of Neotropical lianas (tribe Bignonieae, Bignoniaceae). Our investigation was guided by the long-standing hypothesis that habitat specialization has promoted speciation in Bignonieae, while competition for shared pollinators influences species co-occurrence within communities. We analyzed a geo-referenced database for 94 local communities occurring across the Neotropics. The effect of floral morphological traits and abiotic variables on species co-occurrence was investigated, taking into account phylogenetic relationships. Habitat filtering seems to be the main process driving community assembly in Bignonieae, with environmental conditions limiting species distributions. Differing specialization to abiotic conditions might have evolved recently, in contrast to the general pattern of phylogenetic clustering found in communities of other diverse regions. We find no evidence that competition for pollinators affects species co-occurrence; instead, pollinator occurrence seems to have acted as an “environmental filter” in some habitats.

## Introduction

The importance of species traits for the assembly of communities at local and regional scales is a pervasive topic in ecology [1], [2]. In this context, much attention has been paid to two distinct kinds of processes: environmental filtering, i.e., limits imposed by abiotic conditions, and competition, i.e., biotic interactions arising from common use of limited resources [3]–[5]. While environmental filtering tends to favor co-occurrence of species with similar phenotypes [6]–[8], competition is thought to create phenotypic “evenness” (overdispersion) of species within communities [5], [8], [9]. Thus, these processes are expected to exert opposing effects on the phenotypic structure of communities. The dynamics of trait and lineage evolution are thus relevant to community ecology [5], [7], [10], [11], because depending on whether traits are phylogenetically conserved or not, communities can exhibit significant phylogenetic structure [5], [9], [12], [13]. As these assembly processes are not mutually exclusive, the phenotypic and phylogenetic structure of natural communities is expected to reflect their combined effects [10], [13], [14].

Most studies of plant community assembly have focused on the influences of abiotic aspects or direct interactions among co-occurring plants species [15], although indirect interactions, like those mediated by herbivores or pollinators, have also been shown to be important [15], [16]. In particularly, plant-pollinator interactions have been important for the evolution of floral traits and lineages [17], and consequently for the phenotypic and phylogenetic structure of communities [15], [18]–[20]. Pollinator services have been traditionally viewed as a limiting resource, causing plant competition and species phenotypic repulsion on floral traits and flowering patterns [21]. However, two underappreciated processes that cause phenotypic attraction on floral traits in plant communities have increasingly received empirical support: (i) habitat filtering, with environments determining the pollinators and pollination systems that can persist [15], and (ii) facilitative interaction, in which beneficial pollinator sharing by plant species jointly attracts and/or maintains the populations of pollinators [23], [24].

Here, we evaluate the role of habitat environmental filtering and competition mediated by pollinators for the structure of communities of a large Neotropical clade of flowering plants, the tribe Bignonieae. Bignonieae includes almost half of the species of the family Bignoniaceae (393 out of 827 species), with most of its taxa occupying a variety of habitats across the Neotropics [24], [25]. Most species are lianas, but shrubs are also present in some lineages [26]. The present study owes much inspiration to pioneering research of Bignoniaceae by Gentry [27]–[29]. He observed that within genera, species of Bignoniaceae tend to have allopatric ranges, narrow habitat preferences, and more divergence in vegetative versus floral traits, suggesting allopatric lineage diversification and adaptation to abiotic conditions at broad spatial scales [27], [29]. At local scales, species of Bignoniaceae tend to be self-incompatible, obligatorily outcrossing, and lack natural hybrids, suggesting that competition for pollinators might be an important factor in the assembly of communities [27]–[29]. Indeed, pollination strategy is supposed to have played a key role in the evolution of the tribe, with changes in floral morphology being associated with shifts in pollinator guilds [29], [30]. Up to 20 species of Bignoniaceae have been reported to coexist in natural communities, representing both specialized pollinator guilds (big- to medium-sized bees, bats, hummingbirds, hawkmoths), and more generalist guilds that include butterflies and various smaller insects [27]–[29]. As a result, it has been suggested that Bignoniaceae communities may be saturated in terms of pollinator use, with individual species being pollinated by a different pollinator group in each community at the same time [29].

A molecular phylogenetic study of Bignonieae [31] has cast these hypotheses in a new light, particularly by showing that most of the traditionally recognized genera are not monophyletic and needed a new circumscription. In addition, floral traits previously considered important for taxonomic delimitation were shown to exhibit considerable homoplasy, the phylogenetic signature of labile or recurrent evolution [30], [31]. The objective of the present study is to integrate phylogenetic, environmental, and morphological data with surveys of species co-occurrence to detect the signature of processes driving community assembly in Bignonieae. Specifically, we reformulate Gentry's [29] predictions in an explicit phylogenetic framework, as follows:

### Abiotic predictions

Species from communities that are subject to environmental filtering are expected to show phenotypic attraction in the traits associated with habitat specialization. The expectation of phylogenetic structure in such communities (co-occurrence of close versus distant relatives) depends on whether those traits evolve in a labile or conserved manner [13], [32]. One potential scenario of labile evolution is that species divergence is frequently driven by habitat specialization in allopatry, in which case we would expect species to have narrow abiotic niches and to infrequently co-occur with close relatives, as proposed formerly for Bignoniaceae [29]. In this case, communities will tend to be assembled from more distant relatives, showing phylogenetic evenness or overdispersion [11]. Alternatively, if niche evolution is phylogenetically conservative, communities assembled through environmental filtering will tend to be composed of close relatives and show phylogenetically clustering [13], [32].

### Biotic predictions

If competition for pollinators influences species coexistence, communities should exhibit phenotypic repulsion on floral traits, reflecting diversity in pollination strategies [15], [20]. If floral traits are phylogenetically conserved, communities can be expected to have overdispersed phylogenetic structure (co-occurrence of distant relatives). On the other hand, labile evolution coupled with competition would create a random pattern of phylogenetic community structure [11], [15]. Alternatively, interspecific interactions between co-occurring flowering plants may be facilitative and/or subject to the filtering imposed by the absence of a given pollinator guild [15]. These scenarios would favor phenotypic attraction in plant communities, with the resulting phylogenetic structure being similar to those mediated by traits involved in habitat filtering (see above). As floral traits of Bignonieae have shown contrasting patterns of evolution, with floral morphologies having evolved in a labile way, while other floral features (i.e., size of attractive parts and allometric pattern) exhibit conserved evolution [30], [33], [34], it is hard to predict how such floral traits may contribute to the phenotypic and phylogenetic structure of communities of Bignonieae.

In this study, we used a time-calibrated phylogeny of Bignonieae [35] as an evolutionary framework to investigate these predictions. Particularly, we assess the patterns of species co-occurrence and the associated abiotic variables within the context of their phylogenetic structure, in order to test the specific abiotic predictions. We also evaluate the biotic predictions by assessing the phenotypic structure of floral traits within communities and how this phenotypic structure relates to the phylogenetic and distribution patterns of species.

## Materials and Methods

### Species distribution and communities sampling

We used Alwyn Gentry's transect database as the basis of a dataset of species co-occurrences for Bignonieae (http://www.mobot.org/MOBOT/research/gentry/transect.shtml). In this database, each transect extends 0.1ha, surveyed for the presence and abundance of all plants exceeding 2.5 cm diameter at breast height (dbh). Spatial and environmental variables, such as GIS coordinates and forest physiognomy (i.e., humid or dry forest, savanna), are also recorded. Of the 226 transects available, we restricted our survey to 154 transects located in Central and South America plus Mexico, corresponding to the distribution of Bignonieae (only one species, *Bignonia capreolata*, occurs in the USA). Species of Bignonieae were recorded in 107 transects, of which 18 represented singleton observations and were excluded from further study. Our survey of Gentry's database thus yielded 89 Neotropical transects that contained at least one species of Bignonieae. We supplemented this dataset with additional records of species occurrence and abundance, GIS coordinates, and vegetation physiognomy compiled by one of us (F.R.M.) from floristic inventories. After the exclusion of localities with singletons, this additional dataset yielded five additional sites, substantially improving our sampling of forests in Eastern Brazil (Atlantic rainforest and “Cerrado” areas). A complete account of these 94 localities (hereafter “communities”) is provided in Table S1 (see also Fig. S1).

All communities were classified according to their habitat. We based these “habitat” primarily on the WWF biome classification, which are based on a range of abiotic environmental variables that determine the ecological attributes of an area [36], but subdivided the biome “Tropical and Subtropical Moist Broadleaf Forests” into three separate habitats based in its discrete geographic areas: Central American Moist Forests, Amazonian Moist Forests, and Atlantic Moist Forests. The additional biomes represented in our analyses were: “Deserts and Xeric Shrublands,” “Tropical and Subtropical Coniferous Forests,” “Tropical and Subtropical Dry Forests,” and “Tropical and Subtropical Grasslands, Savannas, and Shrublands” (Table S1). Since species distributions on large spatial scales are related to abiotic environmental conditions, assigning biomes generally corroborates the vegetation physiognomy recorded *in situ* for the communities in our dataset. For example, communities classified as occurring in the Moist Broadleaf Forest biome, were generally described as “tropical moist forest vegetation” or “evergreen/semideciduous forests” in Gentry's transects database, while communities classified as occurring in the Tropical Dry Forest biome were described as “dry forest” [37]. In a few cases, we found discrepancies between the physiognomy classification of the plots in our database and the WWF biome classification. In those cases, we favored the *in situ* classification of habitat, since GIS data can be subject to errors associated with coordinate precision and uncertainty in the models used to predict biomes. Thus, our habitat classification corresponds to a biome-based classification with some changes made in agreement with the vegetation physiognomy reported *in situ* (Table S1).

### Phylogeny

We based our study on a phylogeny of Bignonieae that was reconstructed from chloroplast and nuclear DNA sequences [31], with branch lengths calibrated to time with fossil constraints [35]. This phylogeny includes 106 species of Bignonieae, selected from the 393 species in the tribe in order to cover the range of their morphological and geographical variation [26], [31]. Of the 146 species species encountered in the community dataset, 83 were not included in the molecular phylogeny. To incorporate those additional 83 taxa, we added branches to the tree in polytomous positions corresponding to their most derived morphological synapomorphies [24], [31], with lengths assigned according to ultrametric constraints (Fig. S2). This tree was used for all subsequent analyses.

### Environmental variables

We extracted data for five abiotic variables from the 94 communities represented in our dataset, using the 2.5 arc-second resolution grid available from the WorldClim database (http://www.worldclim.org) and the GIS software ArcMap 9.1 [38]. Variables were chosen for their power to predict species establishment: mean amplitude of monthly temperature, annual amplitude in mean monthly temperature, mean monthly temperature, annual precipitation, and the distribution of precipitation throughout the year (measured using Walsh's [39] index) (Table S1). We also recorded the biome of each community, based on the WWF world terrestrial ecoregion classification [36].

### Floral morphology data

Here, we used the classification of species of Bignonieae according to Gentry's floral morphological “type” [27] derived from an earlier study [30]. In addition, we used quantitative measurements of the 16 floral characters from all four whorls of organs obtained by Alcantara and Lohmann [33]. The morphological dataset used in the present study was complemented with additional information from the species that were found in the plant communities but not sampled in the molecular phylogeny of the group. Floral trait data was recorded as the mean of measurements taken from up to ten specimens per species (see [33] for further details).

### Data analyses

We assessed the influence of phylogeny on species co-occurrence from two perspectives, that of the species and that of the community. From a species perspective, we constructed a matrix of pairwise species co-occurrences, measured by Schoener's [40] index of proportional similarity CI_ih_ = 1−0.5 * (Σ|p_ij_−p_kj_|), where p_ij_ is the proportion of plots j with the occurrence of the species i and p_kj_ is the proportion of plots j where the species k occur. We also constructed a corresponding matrix of pairwise phylogenetic (patristic) distances between species pairs. We then tested for correlation between these matrices using a Mantel test with 9999 permutations [41]. These statistical analyses were carried out using the statistical software R (2004–2008, www.R-project.org). A significant association between these matrices would suggest two opposing scenarios: i) a positive correlation would indicate that distant relatives tend to co-occur, but that closely related species tend not to co-occur, while ii) a negative correlation would indicate the converse.

From a community perspective, we assessed the phylogenetic structure of co-occurring species across sites in order to test whether species in the communities are more or less related than expected by chance. We estimated the net relatedness index (NRI) and the nearest taxon index (NTI) metrics [12] using the software Phylocom ([42]: http://phylodiversity.net/phylocom/). Separate analyses were carried out on site-by-species matrices of presence-absence values and abundance values. The incorporation of species abundance data in the analyses implies that results reflect phylogenetic distances among individuals (abundance-weighted distances) instead of distances among taxa occurring in each sample (see [42] for details). We tested for the significance of NRI and NTI using the null models 0 and 3 available in Phylocom, based on 10,000 randomizations. The null model 0 shuffles the species labels across the phylogeny, randomizing their phylogenetic relationships [42]. The null model 3 uses the independent swap algorithm [43] to create swapped versions of the sample/species matrix, constraining the data to have the same row and column totals of the original matrix. Thus, the number of species per sample and frequency of occurrence of each species across samples are constrained and species co-occurrences are randomized [42]. This null model does not randomize the species abundance values and does not include species from the phylogeny in the randomizations (i.e., the species pool is limited to the species that occur in the matrix). All the analyses were carried out with (i) the whole dataset, which implies that the species pool used to calculate the distributions of null models is formed by all the species present in our sample, and (ii) habitat-specific subsets of samples, where the species pool used to calculate the distributions of null models included only species restricted to the habitat analyzed, in order to detect differences among habitats.

To assess the abiotic variables associated with species occurrences, we carried out a PCA to reduce the five abiotic variables measured for each community to a smaller number of statistically independent variables. For each species, this yielded a set of abiotic PCA scores corresponding to its geographic localities. We quantified the abiotic preferences of a species by calculating the convex hull of points representing its PCA scores. The convex hull is defined as the smallest convex area enclosing a set of points and is a reasonable means of assessing multivariate trait space [6]. This calculation requires at least three points; hence, we excluded the species that only occurred in one or two communities from the dataset. This reduced the number of species from 146 to 76. To test whether species exhibit ecological specialization, i.e., occupy a narrower set of abiotic conditions than expected by chance, we derived a null distribution for the convex hull based on 9999 randomizations of the species-by-locality matrix. These analyses were carried out in the TraitHull program [6], with the total dataset and habitat-specific datasets (i.e., including only the species and communities that occur within a given habitat, see above). During the randomization procedure, we constrained the number of occurrences of each species to be equal to the empirical value. If a species exhibits no abiotic preferences, the convex hull area observed should not fall in the tails of the null distribution; an alternative result would imply that it occupies a smaller or larger region of niche space than expected by chance. We tested this hypothesis with a paired nonparametric two-tailed Wilcoxon signed-ranks test [44].

To evaluate the phylogenetic pattern of abiotic preferences, we calculated the convex hull areas for successively more inclusive clades across the phylogeny. All else being equal, more inclusive clades should have progressively larger convex hulls, owing to cumulative evolutionary divergence of abiotic preferences. If abiotic preferences are phylogenetically conserved (i.e., evolve slowly relative to the rate of cladogenesis), then the convex hulls of closely related species tend to overlap, and the cumulative hull area should be relatively small at recent ancestral nodes. Alternatively, if closely related species are characterized by higher evolutionary divergence in abiotic preferences, the cumulative convex hull area will be relatively larger at recent ancestral nodes. Thus, calculation of convex hull areas for clades of Bignonieae allows us to assess graphically how the disparity in abiotic preferences has accumulated along the phylogeny, without the challenges associated with ancestral state reconstruction.

We also assessed the effect of floral morphology on species co-occurrence from a species perspective and from a community perspective. From a species perspective, we tested for pairwise associations between floral morphology and species co-occurrence using a Mantel test with 9999 permutations. We used the Schoener [40] co-occurrence index to quantify species co-occurrence, and quantified floral differences as the Euclidean distance between species in a multivariate trait space constructed using PCA. From a community perspective, we assessed the intra-community structure of floral morphology, testing whether the floral diversity of species within a community differ from the expectation for communities assembled at random. We calculated the convex hull occupied by co-occurring species, through the PCA scores calculated from floral measurements. As floral morphology and pollinator associations in Bignonieae are also affected by discrete floral traits, we derived scores from Hill-Smith multivariate analyses [45]. All multivariate analyses were carried out in R (2004–2008, www.R-project.org). As floral traits in Bignonieae showed variation in phylogenetic signal [33], we calculated Hill-Smith scores for a series of different trait combinations: (i) all of the 16 continuous traits analyzed; (ii) all the 16 continuous traits analyzed plus the discrete traits “anther position” (included or exserted), “corolla color” (white, red, yellow or magenta), and “nectar guides” (present or absent); (iii) the 16 continuous traits plus the discrete coding of flower morphology; and (iv) separate analyses of the floral trait classes that are evolutionarily conserved and labile, respectively.

To assess how phylogeny is related to floral diversity within communities, we also calculated the phylogenetic diversity [46] of species at each site. To test whether the convex hull of floral traits and the phylogenetic diversity of co-occurring species are different from communities assembled at random, we used the null model implemented in TraitHull [6], which generates a null distribution of 9999 communities with a given number of species, with species sorting from the original species pool. We used a modified version of the TraitHull script that included the estimation of phylogenetic diversity of communities given a tree (available from the authors upon request). Two-tailed Wilcoxon signed-ranks tests were used to test whether observed convex hulls differed from the null distribution [44]. A convex hull in the high tail of the null distribution would indicate that species differ in floral morphology more than expected by chance, while a convex hull in the low tail of the null distribution would indicate that species are more similar than expected [6].

To allow for comparisons among communities with different numbers of species, we ranked the observed values of convex hull and phylogenetic diversity based on the null distribution generated for each distinct value of community species richness. This ranking was used to compare the pattern of morphological and phylogenetic diversity among communities from different habitats. The correlation between ranked phylogenetic diversity and convex hull values were tested through the Spearman's coefficient of correlation [44]. Estimates of convex hull and phylogenetic diversity for communities located in different habitats using the “habitat species pools” instead of the total species pool were also carried out in order to account for regional differences on species distribution, as might arise if species of Bignonieae are restricted in their distributions by environmental conditions like predicted in the predominance of filtering.

## Results

### Phylogeny and species distribution

There was no correlation between the paired species co-occurrence index and the paired phylogenetic distance among species (Mantel's test: r = −0.002; p = 0.555). In general, there was no phylogenetic structure in the communities analyzed, with only a few values of NRI and NTI being statistically significant (Table S1). The same general pattern was observed for both the analyses using the total species pool and using habitat-specific species pools (data not shown); for convenience, we report here only the results for the total species pool (Table S1). The patterns observed by including abundance data did not differ from those obtained with presence/absence data; thus, we report the details of the former. Most NRI and NTI values were negative (NRI: 54 out the 94 communities with null model 0, and 75 communities with the null model 3; NTI: 62 communities with the null model 0, and 53 communities with the null model 3). The communities that showed significant NRI with null model 0 were: B012, C020, C038, M11, and S143 (Table S1). Only B010 showed a positive value of NRI, indicating that the relatedness of individuals within that community was lower than expected. With null model 3, significant NRI were found in the communities C025, C038, C058, R133, T154, and Y166. C058 and R133 showed higher values of NRI than expected, while the others had lower values. NTI were significant for the communities C038, D063, M111, and T155 with the null model 0, being positive only in D063. With the null model 3, only D063 and M111 showed significant values of NTI, which were positive and negative, respectively.

### Abiotic preferences and habitat specialization

**Figure 1:**
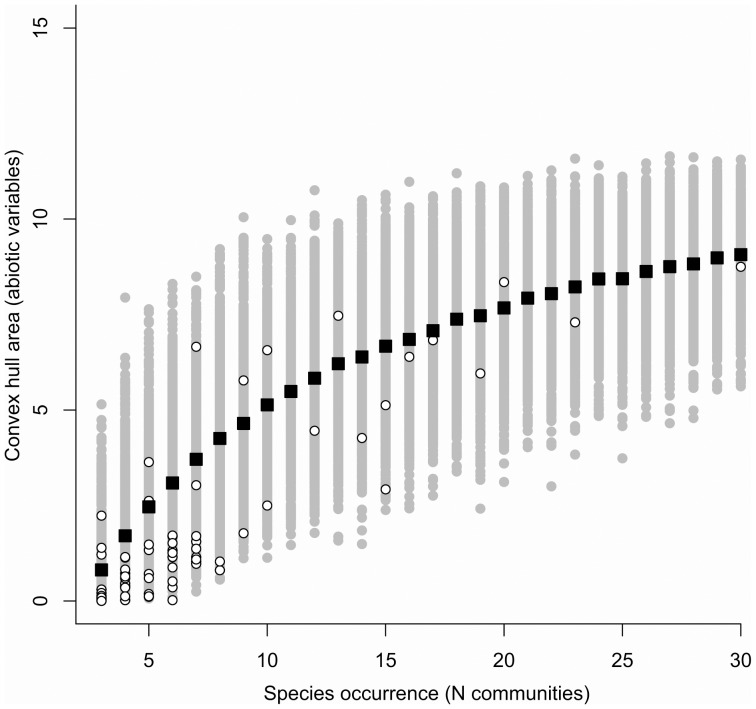
Convex hull area size of 76 species of Bignonieae from their abiotic variables. Convex hull were estimated from the two PC axes scores. Species occurrence indicates the number of communities in which a species was recorded. Open circles represent the observed values of convex hull. Grey circles represent the estimated null distribution of convex hull (see text). Black squares show the mean of the null distribution calculated from each species occurrence number.

The two PCA axes used to estimate the abiotic convex hull occupied by species of Bignonieae explained 45.8% and 26.5%, respectively (data not shown), indicating that most variation in the abiotic variables analyzed was included in the convex hull estimates. Species of Bignonieae occupied lower convex hulls (i.e., narrower ranges of abiotic conditions) than expected by chance (Wilcoxon test: V = 154; p = 0.0016; Fig. 1). These results did not differ from the analyses carried out with habitat-specific subsets (data not shown).

**Figure 2:**
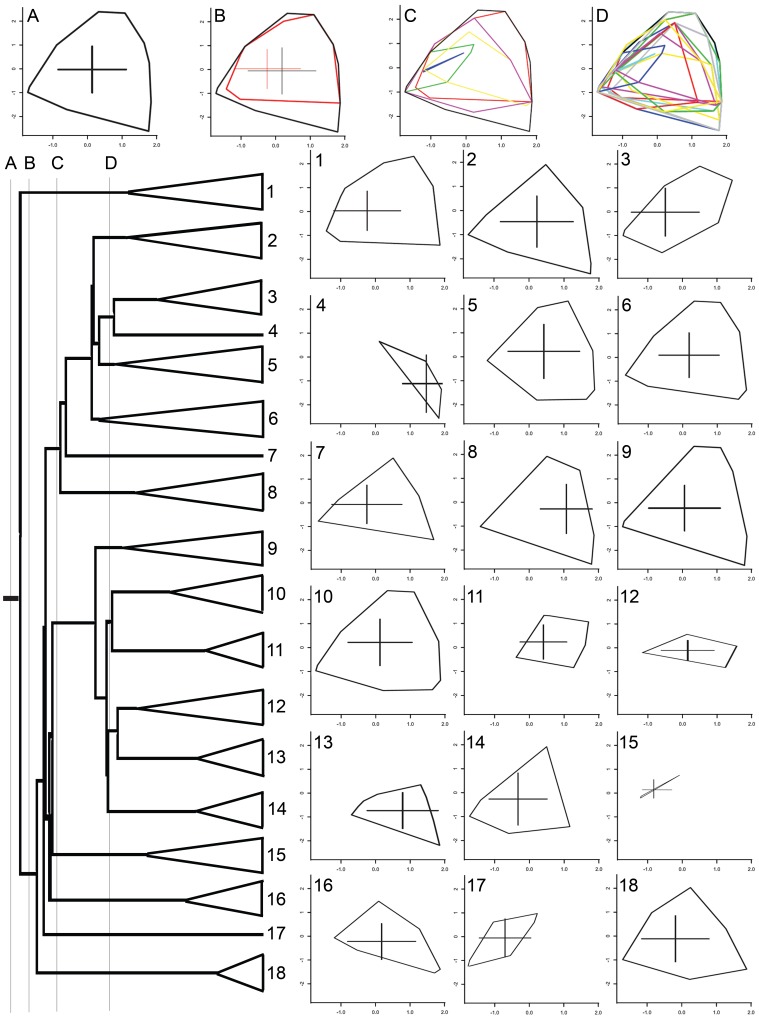
Size of the abiotic variables hyperspace occupied for the most including nodes across the phylogeny of Bignonieae. Graphics indicate the convex hull areas delimited by the abiotic preferences of the species included in each genus (identified by numbers) and more inclusive clades (identified by letters). Total size of convex hulls for individual species and branches of the phylogeny are shown in the Fig. S3. 1. *Adenocalymma*. 2. *Amphilophium*, 3. *Anemopaegma*. 4. *Pyrostegia*. 5. *Mansoa*. 6. *Bignonia*. 7. *Callichlamys*. 8. *Dolichandra*. 9. *Tanaecium*. 10. *Fridericia*. 11. *Xylophragma*. 12. *Cuspidaria*. 13. *Tynanthus*. 14. *Lundia*. 15. *Pachyptera*. 16. *Pleonotoma*. 17. *Martinella*. 18. *Stizophyllum*.

Convex hull calculated for clades in the phylogeny concentrated the most differences amongst species within genera instead of between genera, with lowest divergences in convex hull area occurring in the most inclusive clades (Fig. 2, Fig. S3).

### Floral morphology and species co-occurrence

Pairwise floral divergence between species of Bignonieae was not significantly related to co-occurrence (Mantel's test: r = 0.0025; p = 0.452). In general, floral diversity observed in communities of Bignonieae did not differ from the null expectation that communities are assembled randomly (Wilcoxon test: V = 79; p = 0.31). Analyses carried out with the total species pool and with the habitat species pool did not differ; thus, we only describe the results derived from the former (Fig. S4). Similarly, analyses carried out with different subsets of floral traits also showed similar results; for convenience, we report the results obtained with one combination of traits (the second listed in Materials and Methods). Hill-Smith ordination of this dataset indicated that the two first axes explain 52.1% and 11% of the variation of floral traits, respectively (data not shown).

**Figure 3:**
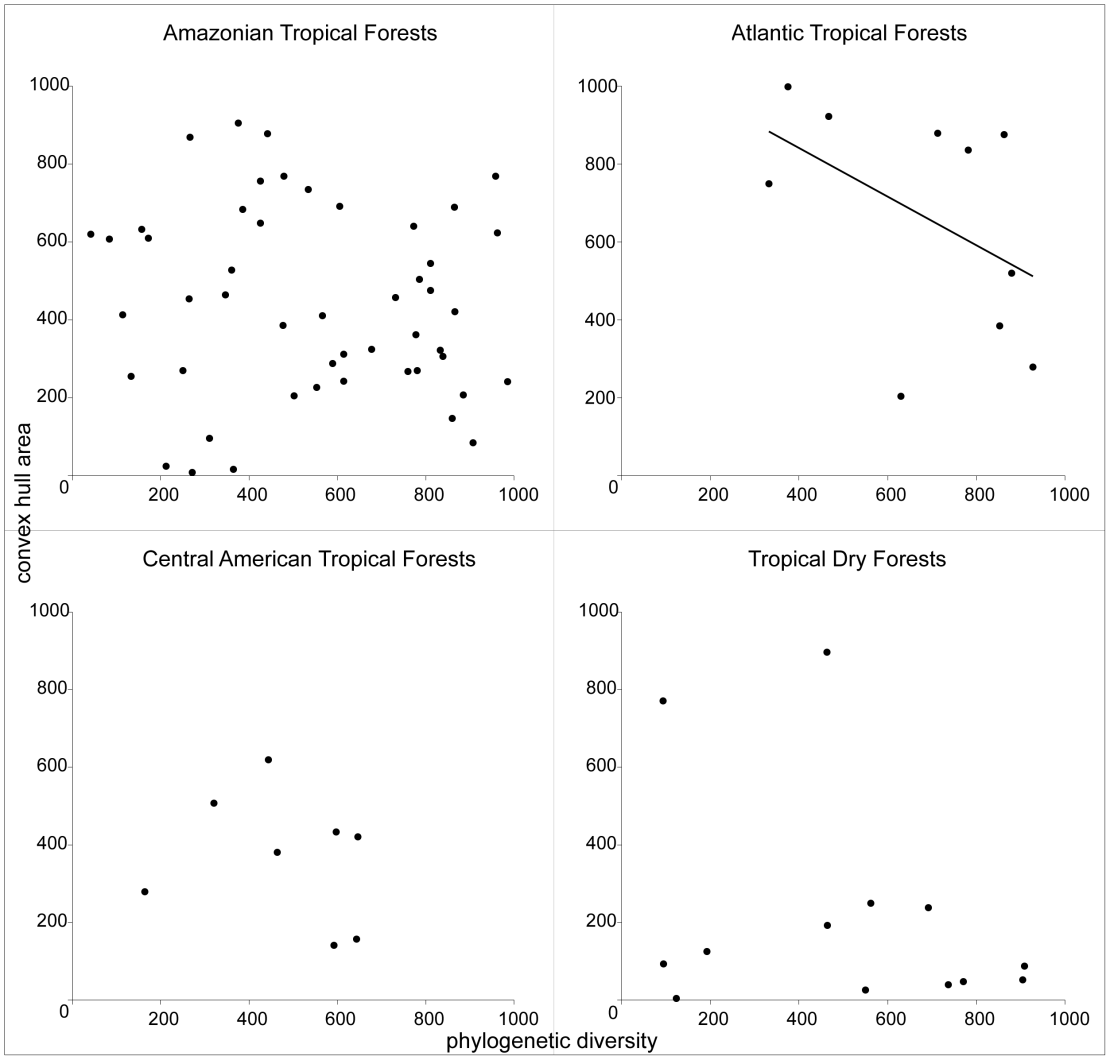
Floral diversity *versus* phylogenetic diversity in communities of Bignonieae in different habitats. Ranked values of (i) convex hull area, representing the morphological floral diversity, and (ii) phylogenetic diversity, calculated as the sum of phylogenetic branch lengths of the species in each community. The recorded points represent each of the communities located at the following habitats: Amazonian Tropical Forests; Atlantic Tropical Forests; Central American Tropical Forests; and Tropical Dry Forests.

Rank-based correlation analysis of floral convex hull area and phylogenetic diversity did not reveal general significant associations (Spearman rho = −0.043; p = 0.347; Fig. S5). However, visual inspection of the results revealed notable patterns in three out the six habitats analyzed. Communities in the Atlantic Moist Forests had higher diversity of floral morphology than the other biomes (Wilcoxon test: V = 222; p = 0.007), with marginal evidence for a negative correlation with phylogenetic diversity (Spearman rho = −0.612; p = 0.066; Fig. 3A). In contrast, communities from Tropical Dry Forests had relatively low diversity of floral morphology (Wilcoxon test: V = 811.5; p = 0.0034; Fig. 3B), a pattern also exhibited by the only two communities sampled in the Tropical Savannas (Fig. S5). As far as phylogenetic diversity is concerned, only Tropical Savannas were notably different by presenting lower diversity than the other habitats (Fig. S5).

## Discussion

In this paper, we investigated the structure of communities of a Neotropical clade of lianas, bringing phylogeny to bear on questions of how evolutionary patterns of species' traits might influence community assembly. We were particularly motivated by Gentry's [29] predictions that (i) species are specialized to abiotic conditions, and that (ii) communities are saturated in terms of pollination niche. A primary result from our study is that communities of Bignonieae are not phylogenetically structured, i.e., close relatives do not co-occur more than expected by chance or less frequently than expected by chance. This suggests that opposing assembly processes favoring close and distant relatives, respectively, may be at work [14]. This finding is also consistent with the hypothesis that competition among species (e.g., for pollinators) is coupled with labile and presumably adaptive evolution of traits that mediate their competitive interactions [5], [11]. The available metrics for characterizing phylogenetic community structure have low power to detect evenness/overdispersion, i.e., the tendency of distant relatives to co-occur more expected by chance [13]. However, the lack of resolution at the terminals of the phylogeny is not expected to substantially affect detection of phylogenetic structure, but would instead contribute to a signal of random phylogenetic structure [47]. Nevertheless, our results conclusively reject the expectation that tropical communities with large regional species pools exhibit phylogenetic clustering [11], [48].

Lack of phylogenetic clustering was persistent in both habitat-specific (regional) and total (continental) species pools. This result differs from the general trend toward increased phylogenetic clustering at larger geographical scales, or outside the “Darwin-Hutchinson zone” (reviewed in [48]). This increased clustering is expected on continental scales, as a signature of biogeographic processes that reflect dispersal abilities of clades [49]. In Bignonieae, the lack of phylogenetic structure at regional and continental species pools suggests that limited dispersal and/or significant biogeographic barriers have not had major effects on local community structure. In addition, our data set shows that different species from several lineages are broadly distributed and seemingly able to disperse and persist across ecological zones and biomes, suggesting labile evolution of abiotic tolerances [50]. In contrast to this niche-based perspective, the lack of phylogenetic community structure might be attributable to neutral processes of community assembly [51], [52]. However, the difficulty in ruling out contrasting niche-based processes that operate on different scales, and the uncertainty of appropriate null models and species pools, challenge this interpretation [32]. If neutral processes were indeed the prevailing force, one would expect to find no signals concerning habitat preferences. However, we point out that habitat preferences of species and floral traits distribution contribute to the distribution of Bignonieae species across habitats (see below).

Species of Bignonieae tend to occupy a limited portion of the potential convex hull space predicted by their abiotic variables, compared to a null model in which species can occupy any of the communities sampled (Fig. 1). This pattern suggests that, with few exceptions (e.g., *Dolichandra unguis-cati*, *Stizophyllum riparium*, and *Tanaecium pyramidatum*), most species are characterized by specialization to a restricted set of abiotic conditions. Quantification of the convex hull for successively inclusive clades in the phylogeny of Bignonieae shows greater evolutionary divergence at more recent nodes and less divergence at deeper phylogenetic nodes (Fig. 2). Thus, habitat specialization seems to have evolved *within* more recent clades of the phylogeny, like generic or sub-generic clades. It also suggests that similar abiotic preferences have convergently evolved in different clades. The specialization of closely related species to different abiotic conditions corroborates part of Gentry's [29] hypothesis. The second prediction of this hypothesis, that speciation is driven by allopatric specialization, would lead to a negative association among species relatedness and their co-occurrence, which we did not find here. However, most variation in species preference attributes occurs at infra-generic level, for which we have not enough phylogenetic resolution (Fig. S2). Thus, this second prediction still remains to be tested with a phylogeny resolved below the genus level. We did not assess here the effect of potential bias in the geographic locations of communities (i.e., most are located in the Western Amazon; Fig. S1). However, most species of Bignonieae are exclusively Amazonian [24], [26]. In addition, we have likely sampled the most common species instead of the rarest ones, suggesting that increased sampling might not change the general pattern of abiotic specialization found here.

The attraction of species possessing traits that enable habitat occupancy characterizes the process of habitat filtering [7], [12]. Unfortunately, we have no specific information about the traits in Bignonieae that are functionally associated with the environmental variables we have studied here. In fact, few large-scale tests of coexistence theories in tropical forests have explicitly examined the ecological strategy of co-occurring species [53]. Those studies revealed pervasive habitat specialization affecting species coexistence even in diverse systems [11], [14], [54]. Moreover, important plant functional traits show evidence of phylogenetic conservatism, as leaf traits [55], wood density [56], and resource allocation patterns [57]. The combination of trait conservatism and environmental filtering has been presumed to account for phylogenetic clustering in many plant communities [48]. Environmental filtering can also cause phylogenetic overdispersion if traits that are important for habitat specialization are labile, with close relatives specializing to different environments [5], [9], [58]. Further studies focusing on the functional traits coupled with infrageneric phylogenies are needed to evaluate whether this is the case in Bignonieae. This topic is particularly exciting given the relatively rapid and recent evolution suggested for environmental specialization and the increasing changes in natural habitats and global climatic conditions.

There were no effects of floral similarity on species co-occurrence and intracommunity structure, rejecting the hypothesis of saturation by pollinators, to the extent that our measurements of floral morphology accurately reflect pollination mode in Bignonieae [27], [30]. This pattern remains even when analyses are carried out with habitat species pools, similar to the pattern found for phylogenetic distance among species. The frequent shifts in floral morphology and the low phylogenetic signal in floral form encountered in Bignonieae were previously interpreted as being indicative of competitive displacement caused by competition for pollinators [33]. Our results imply, however, that the saturation caused by competition by pollinators might have had minor effects on the community assembly of Bignonieae. Nevertheless, there are significant differences in the overall floral diversity of communities located in different habitats. More specifically, less floral diversity than expected by random assemblage found in communities located in the Tropical Dry Forests and Tropical Savannas were detected, while higher diversity was found in Atlantic Tropical Forests.

Similarly to how species' abiotic preferences influences local community structure, habitat specific differences in pollinator pools could directly influence the floral diversity of communities [15]. The local pollinator community can act directly as a biotic filter in an area without suitable pollinators, or indirectly, if the physical environment (i.e., light spectrum, climate, water availability) influences plant-pollinator interactions, determining which pollination systems can persist [15]. Moreover, the occurrence of facilitative interactions between plants that share pollinator guilds has received increased evidence [22], [23]. Both pollinator-driven filtering and plant-driven facilitation could create the pattern we found here. Species of Bignonieae are obligate out-crossers and depend on animals for pollination; hence, the absence or rarity of a given pollinator guild in an area would limit species establishment. Finally, correlations among floral morphology and specific vegetative traits associated with abiotic specialization could create differences in floral diversity among habitats, which is also in agreement with the concept of indirect habitat filtering [6], [11], [59].

Founder-effect colonization of areas by relatively few lineages within Bignonieae may also explain the low morphological diversity in the two communities located in Tropical Savannas, which also showed lower phylogenetic diversity than the other habitats. Evidence indicates that Bignonieae originated in the Atlantic rainforest area and diversified in the Amazon Basin [35], with few species evolving the ability to colonize savannas [26]. The diversification of restricted lineages within Bignonieae in this habitat may not have allowed the accumulation of phylogenetic and morphological diversity compared with that occurred in humid forests.

On the other hand, we did not find any indication of phylogenetic or biogeographic structure in species distributions of Bignonieae species in Tropical Dry Forests. These habitats have already been reported as subject to strong phylogenetic and geographic structure [60]. Instead, our data support the hypotheses that strong environmental filtering may have contributed to the assemblage of Tropical Dry Forests communities, at least in terms of plant-pollinator interactions and their associated morphological traits. Dry areas are known to have the highest levels of bee diversity [61], and most species of Bignonieae have an open-mouthed flower morphology associated with bee pollination [27], [30]. This Anemopaegma-type flower is the prevalent morphology within Tropical Dry Forests, and we hypothesize that the predominance of pollination by bees in those areas has limited the occurrence of species with different pollinator vectors. In addition, this floral type was identified as the ancestral morphology of Bignonieae flowers and is widespread between the genera [30]. This would account for the absence of relationship between phylogenetic and morphological diversity in those communities.

The higher morphological diversity found in communities of Atlantic Tropical Forests than in the other habitats has a marginally negative association with their phylogenetic diversity, a trend opposite to the pattern observed in the Tropical Savannas. Evidence indicates that Bignonieae likely originated ca. 50My ago in the same geographical region that is currently occupied by Atlantic Tropical Forests of Brazil [35]. This long-time occupancy and the old age of tropical humid forests would lead to the accumulation of morphological diversity, while the recurrent invasions and diversification at the Amazon Basin would lead to the negative association between phylogeny and morphological diversity observed. Notably, despite the fact that Atlantic Forests are less diverse in their hummingbird fauna than Andean and Amazon Forests, this biome is as diverse as the Andean and Amazonian forests in terms of the number of plant species pollinated by hummingbirds [62]. The morphology associated with hummingbird pollination is the second most common floral form within Bignonieae species, and the most homoplastic one [30]. The suggestion that pollinator faunas have filtered species occurrence across different habitats has important implications for conservation considering the recent worldwide decline of pollinators [15], [63], [64]. Despite the lack of precise estimates of pollinator diversity on these habitats, these broad patterns represent an intriguing avenue of investigation into the causal relationship between morphology and pollinator diversity in communities of Bignonieae in different habitats.

### Conclusion

Our results allowed us to reject the hypothesis that competition for pollinators causes floral saturation and represents a major factor structuring the communities of Bignonieae. Nevertheless, they corroborate Gentry's [27], [29] hypothesis that pollination mode may be an important determinant of Bignoniaceae occurrence. We speculate that the specialization to abiotic conditions in this group must have evolved recently, although we did not find the patterns expected by specialization occurred in allopatry, which corroborate only partially the former hypothesis of habitat specialization [29]. Our results differ from the general pattern revealed by most studies of phylogenetic community structure, which report phylogenetic clustering of local communities within larger species pools (reviewed in [48]). Vamosi et al. [48] suggested a common role for habitat filtering coupled with species conserved functional traits, which is opposite to the pattern of evolutionary lability we suggest here. Specialization to abiotic conditions and divergence in floral diversity among habitats suggest a niche-based filtering, concurring with other reports available for tropical forests that suggest that neutral forces may not be sufficient to explain species distributions and the maintenance of diversity in tropical forests [14], [53], [54].

## Supporting Information

Figure S1 **Distribution of the 94 communities included in this study.** See Table S1 for specific details.
(PDF)

Figure S2 **Phylogeny of Bignonieae used in this study, with the manual inclusion of 83 species in 22 polytomies representing genera or infra-generic clades in a time calibrated tree originally containing 106 species, of which 63 species originally included were kept.** Branch lengths are represented proportional to time (see text).
(PDF)

Figure S3 **Total convex hull size of the 76 species of Bignonieae and of the most inclusive clades of the phylogeny.**
(PDF)

Figure S4 **Convex hull area estimates for 86 communities of Bignonieae that contain more than 2 species, from the two PC axes obtained from the floral morphology variables included in this study.** Species richness indicates the number of species sampled in that community. Open circles represent the observed values of convex hull, and grey circles represent the estimated null distribution of convex hull (see text). Dashed line shows the observed convex hull tendency, while black squares show the mean of the null distribution calculated from each species occurrence number.
(PDF)

Figure S5 **Ranked distribution of the observed phylogenetic diversity and convex hull area (calculated from the flower morphological scores) of the species of Bignonieae occurring in the communities studied.** Different points represent communities located in different habitats: AMA  =  Amazonian Moist Forests; ATL  =  Atlantic Moist Forests; CEN  =  Central American Moist Forests; DRY  =  Tropical and Subtropical Dry Forests; DXS  =  Deserts and Xeric Shrublands; SAV  =  Tropical and Subtropical Grasslands, Savannas, and Shrublands.
(PDF)

Table S1 **Complete list of the 94 communities studied.** Location  =  politic name of locality and country, GEO  =  geographic coordinate, N  =  number of species in the community, MMA  =  annual Mean of Monthly temperature Amplitude (estimated as the average of the values of monthly temperature amplitude), AMMT  =  annual Amplitude in the Mean monthly Temperature (estimated from the difference between the highest and lowest mean monthly temperature), AMT  =  annual mean temperature, AP  =  annual precipitation, Walsh's index  =  precipitation distribution along the year, Biome  =  following WWF's classification, Habitat  =  based on WWF's biomes and on local physiognomy vegetation (see text), Null model 0  =  shuffle species in the tips of phylogeny, Null model 3 =  independent swap algorithm, NRI (r) and NTI (r)  =  Net Relatedness Index and Nearest Taxon Index, respectively, with the respective number of randomizations lower than the observed. Significant values (higher than 975 or lower than 25) indicate p<0.05.
(PDF)
