# Correction: The Effect of Phylogeny, Environment and Morphology on Communities of a Lianescent Clade (Bignonieae-Bignoniaceae) in Neotropical Biomes

**DOI:** 10.1371/journal.pone.0102720

**Published:** 2014-07-08

**Authors:** The PLOS ONE Staff

There are errors in the Funding section. The correct funding information is as follows: This study was supported by FAPESP (doctoral grant 2006/59916-0); CCSD-Missouri Botanical Garden (Elizabeth E. Bascom Fellowships for Latin American Female Botanists); a FAPESP-NSF-NASA Biota/Dimensions of Biodiversity Grant (FAPESP 12/50260-6); and a CNPq Pq-1C Grant to LGL. The funders had no role in study design, data collection and analysis, decision to publish, or preparation of the manuscript.
