# Supplementary material for: The Effect of Phylogeny, Environment and Morphology on Communities of a Lianescent Clade (Bignonieae-Bignoniaceae) in Neotropical Biomes
Source: PLoS One. 2014 Mar 3;9(3):e90177. doi: 10.1371/journal.pone.0090177 (PMC3940842; doi:10.1371/journal.pone.0090177)

Figure S1. Distribution of the 94 communities included in this study. See Table S1 for specific details.

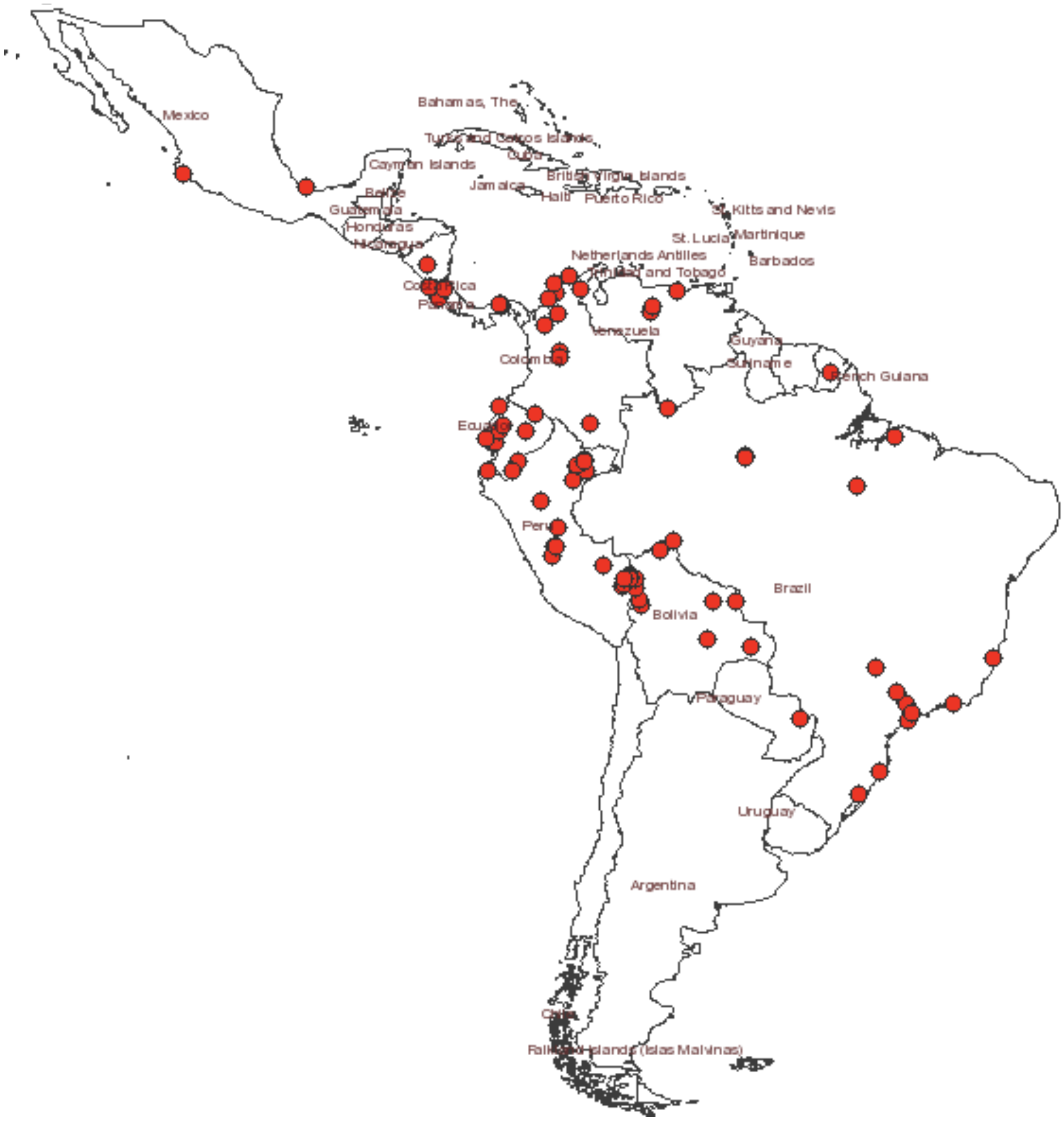

Supplement: Figure S1 — Distribution of the 94 communities included in this study. See Table S1 for specific details. (PDF) [file pone.0090177.s001.pdf]
