# Supplementary material for: The Effect of Phylogeny, Environment and Morphology on Communities of a Lianescent Clade (Bignonieae-Bignoniaceae) in Neotropical Biomes
Source: PLoS One. 2014 Mar 3;9(3):e90177. doi: 10.1371/journal.pone.0090177 (PMC3940842; doi:10.1371/journal.pone.0090177)

Figure S2. Phylogeny of Bignonieae used in this study, with the manual inclusion of 83 species in 22 polytomies representing genera or infra-generic clades in a time-calibrated tree originally containing 106 species, of which 63 species originally included were kept. Branch lengths are represented proportional to time (see text).

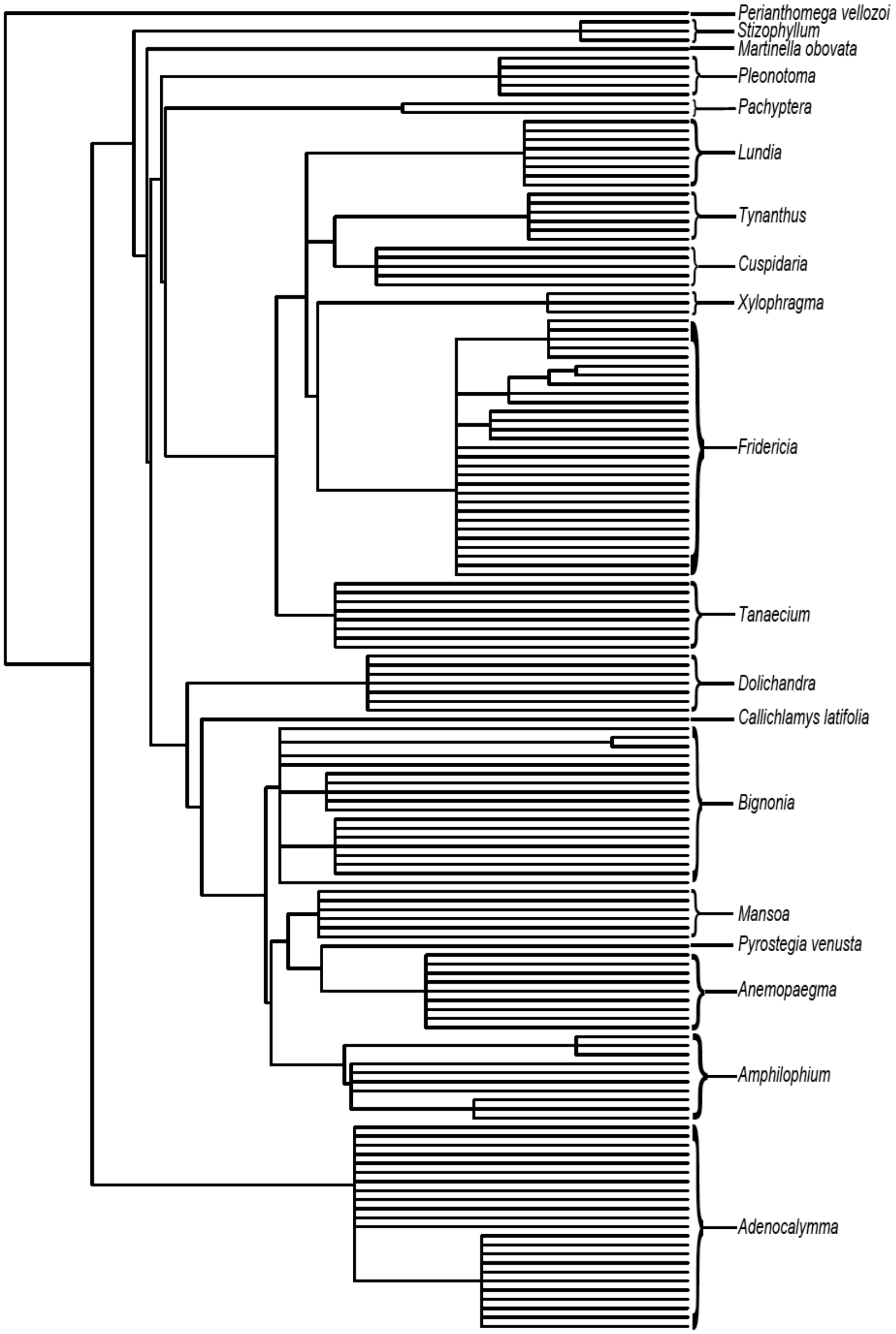

Supplement: Figure S2 — Phylogeny of Bignonieae used in this study, with the manual inclusion of 83 species in 22 polytomies representing genera or infra-generic clades in a time calibrated tree originally containing 106 species, of which 63 species originally included were kept. Branch lengths are represented proportional to time (see text). (PDF) [file pone.0090177.s002.pdf]
