# Supplementary material for: The Effect of Phylogeny, Environment and Morphology on Communities of a Lianescent Clade (Bignonieae-Bignoniaceae) in Neotropical Biomes
Source: PLoS One. 2014 Mar 3;9(3):e90177. doi: 10.1371/journal.pone.0090177 (PMC3940842; doi:10.1371/journal.pone.0090177)

Figure S3. Total convex hull size of the 76 species of Bignonieae and of the most inclusive clades of the phylogeny.

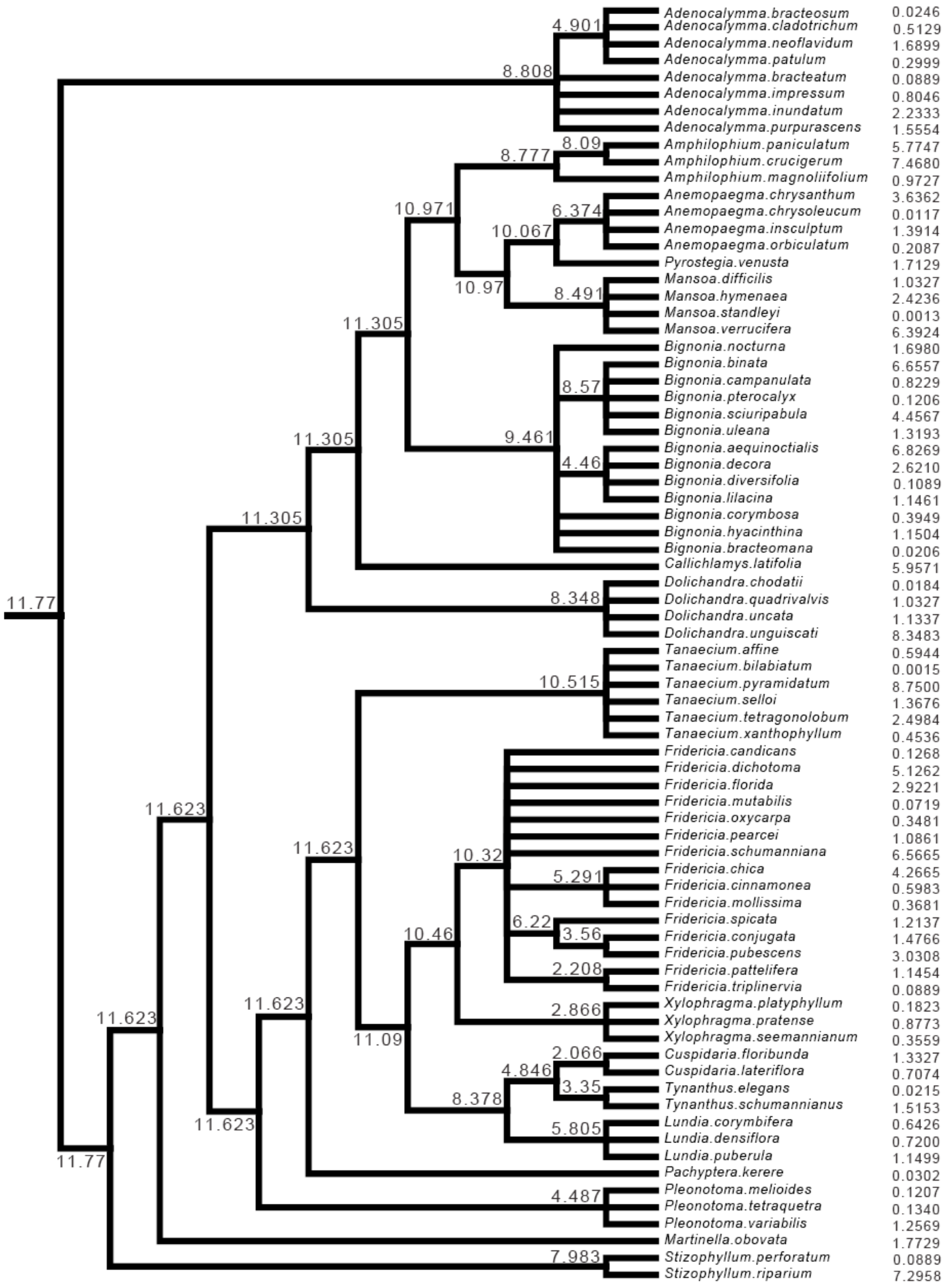

Supplement: Figure S3 — Total convex hull size of the 76 species of Bignonieae and of the most inclusive clades of the phylogeny. (PDF) [file pone.0090177.s003.pdf]
