# Supplementary material for: The Effect of Phylogeny, Environment and Morphology on Communities of a Lianescent Clade (Bignonieae-Bignoniaceae) in Neotropical Biomes
Source: PLoS One. 2014 Mar 3;9(3):e90177. doi: 10.1371/journal.pone.0090177 (PMC3940842; doi:10.1371/journal.pone.0090177)

Figure S4. Convex hull area estimates for 86 communities of Bignonieae that contain more than 2 species, from the two PC axes obtained from the floral morphology variables included in this study (see text). Species richness indicates the number of species sampled in that community. Open circles represent the observed values of convex hull, and grey circles represent the estimated null distribution of convex hull (see text). Dashed line shows the observed convex hull tendency, while black squares show the mean of the null distribution calculated from each species occurrence number.

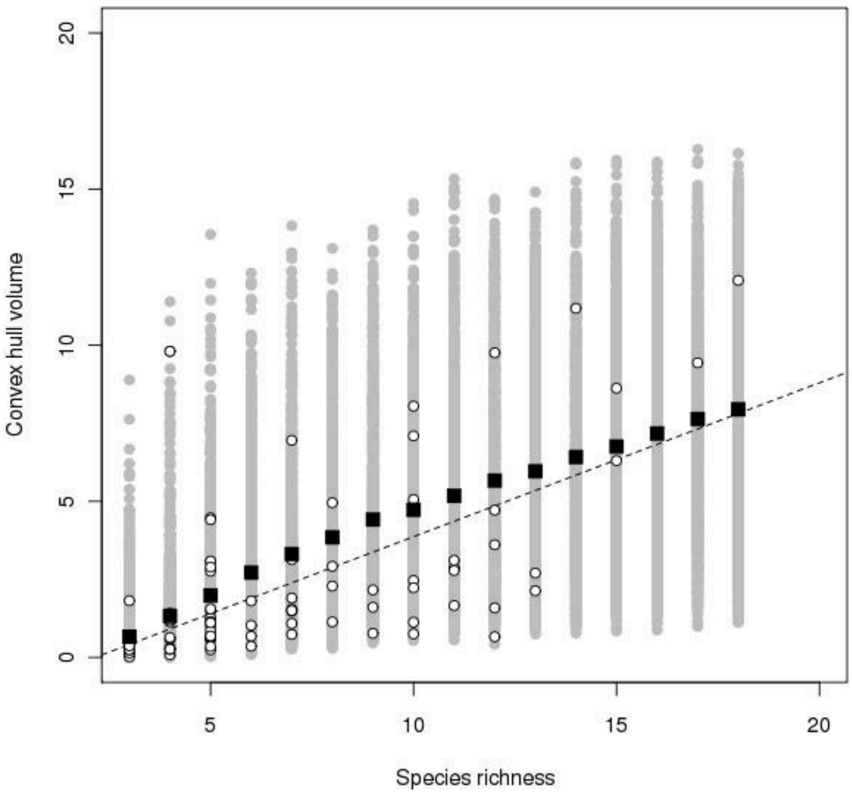

Supplement: Figure S4 — Convex hull area estimates for 86 communities of Bignonieae that contain more than 2 species, from the two PC axes obtained from the floral morphology variables included in this study. Species richness indicates the number of species sampled in that community. Open circles represent the observed values of convex hull, and grey circles represent the estimated null distribution of convex hull (see text). Dashed line shows the observed convex hull tendency, while black squares show the mean of the null distribution calculated from each species occurrence number. (PDF) [file pone.0090177.s004.pdf]
