# Supplementary material for: The Effect of Phylogeny, Environment and Morphology on Communities of a Lianescent Clade (Bignonieae-Bignoniaceae) in Neotropical Biomes
Source: PLoS One. 2014 Mar 3;9(3):e90177. doi: 10.1371/journal.pone.0090177 (PMC3940842; doi:10.1371/journal.pone.0090177)

Figure S5. Ranked distribution of the observed phylogenetic diversity and convex hull area (calculated from the flower morphological scores) occupied by the species of Bignonieae occurring in the 94 communities studied. Different points represent communities located in different habitats: AMA = Amazonian Moist Forests; ATL = Atlantic Moist Forests; CEN = Central American Moist Forests; DRY = Tropical and Subtropical Dry Forests; DXS = Deserts and Xeric Shrublands; SAV = Tropical and Subtropical Grasslands, Savannas, and Shrublands.

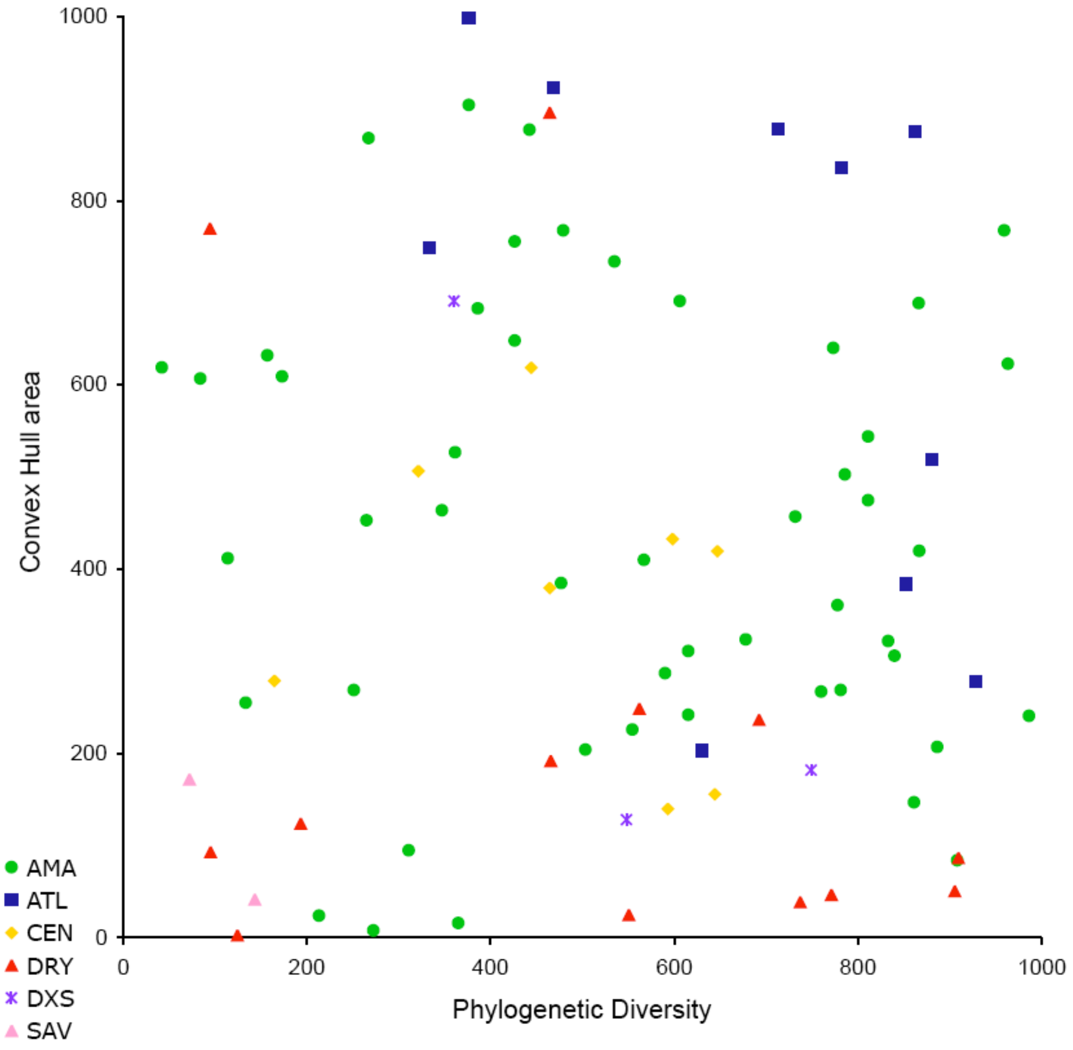

Supplement: Figure S5 — Ranked distribution of the observed phylogenetic diversity and convex hull area (calculated from the flower morphological scores) of the species of Bignonieae occurring in the communities studied. Different points represent communities located in different habitats: AMA = Amazonian Moist Forests; ATL = Atlantic Moist Forests; CEN = Central American Moist Forests; DRY = Tropical and Subtropical Dry Forests; DXS = Deserts and Xeric Shrublands; SAV = Tropical and Subtropical Grasslands, Savannas, and Shrublands. (PDF) [file pone.0090177.s005.pdf]
