# Supplementary material for: The Effect of Phylogeny, Environment and Morphology on Communities of a Lianescent Clade (Bignonieae-Bignoniaceae) in Neotropical Biomes
Source: PLoS One. 2014 Mar 3;9(3):e90177. doi: 10.1371/journal.pone.0090177 (PMC3940842; doi:10.1371/journal.pone.0090177)
Supplement: Table S1 — Complete list of the 94 communities studied. Location = politic name of locality and country, GEO = geographic coordinate, N = number of species in the community, MMA = annual Mean of Monthly temperature Amplitude (estimated as the average of the values of monthly temperature amplitude), AMMT = annual Amplitude in the Mean monthly Temperature (estimated from the difference between the highest and lowest mean monthly temperature), AMT = annual mean temperature, AP = annual precipitation, Walsh's index = precipitation distribution along the year, Biome = following WWF's classification, Habitat = based on WWF's biomes and on local physiognomy vegetation (see text), Null model 0 = shuffle species in the tips of phylogeny, Null model 3 = independent swap algorithm, NRI (r) and NTI (r) = Net Relatedness Index and Nearest Taxon Index, respectively, with the respective number of randomizations lower than the observed. Significant values (higher than 975 or lower than 25) indicate p<0.05. (PDF) [file pone.0090177.s006.pdf]

Table S1. Complete list of the 94 communities studied. Location = politic name of locality and country, GEO = geographic coordinate, N = number of species in the community, MMA = annual Mean of Monthly temperature Amplitude (estimated as the average of the values of monthly temperature amplitude), AMMT = annual Amplitude in the Mean monthly Temperature (estimated from the difference between the highest and lowest mean monthly temperature), AMT = annual mean temperature, AP = annual precipitation, Walsh's index = precipitation distribution along the year, Biome = following WWF's classification, Habitat = based on WWF's biomes and on local physiognomy vegetation (see text), Null model 0 = shuffle species in the tips of phylogeny, Null model 3 = independent swap algorithm, NRI (r) and NTI (r) = Net Relatedness Index and Nearest Taxon Index, respectively, with the respective number of randomizations lower than the observed. Significant values (higher than 975 or lower than 25) indicate $p < 0.05$.

Code	Location	GEO	N	MMA (°C)	AMMT (°C)	AMT (°C)	AP (mm)	Walsh’s index	Biome	Habitat	Null model 0		Null model 3	
											NRI (r)	NTI (r)	NRI (r)	NTI (r)
A002	Allpahuayo (Peru)	0°55’33.9” 46°40’19.8”	6	9.6	1.6	26.3	2772	20.0	Tropical and Subtropical Moist Broadleaf Forests	AMA	-0.396 (421)	-0.844 (207)	-1.126 (63)	-0.341 (369)
A005	Cerros de Amotape (Peru)	04°09’ 80°37’	5	12.0	2.1	20.3	755	-11.0	Tropical and Subtropical Dry Broadleaf Forests	DRY	-0.011 (534)	-0.098 (421)	-0.866 (195)	-0.076 (493)
A007	Araracuara (Colombia)	00°25’ 72°20’	5	8.7	2.4	26.7	2921	19.5	Tropical and Subtropical Moist Broadleaf Forests	AMA	-0.865 (198)	-1.449 (72)	-1.228 (37)	-1.057 (149)
B010	Belém-Mocambo (Brazil)	01°30’ 47°59’	7	9.4	1.2	26.7	2384	11.5	Tropical and Subtropical Moist Broadleaf Forests	AMA	-0.243 (457)	1.559 (927)	-0.714 (215)	1.469 (908)
B012	Blohm Ranch (Venezuela)	08°34’ 67°35’	5	10.2	3.4	27.4	1377	-0.5	Tropical and Subtropical Grasslands, Savannas, and Shrublands	SAV	2.817* (992)	1.796 (952)	1.616 (923)	2.054 (961)
B015	Boracéia (Brazil)	23°23’ 46°00’	2	9.6	6.7	18.7	1400	0.5	Tropical and Subtropical Moist Broadleaf Forests	ATL	0.057 (711)	0.057 (711)	-0.722 (293)	0.023 (695)
C018	Cabeza de Mono (Peru)	10°20’ 75°18’	5	12.1	1.5	22.9	2495	13.5	Tropical and Subtropical Moist Broadleaf Forests	AMA	0.327 (705)	0.604 (746)	-0.36 (440)	0.65 (761)
C019	Calabatea (Bolivia)	14°65’ 68°20’	4	9.3	3.4	20.2	1352	0.5	Tropical and Subtropical Moist Broadleaf Forests	AMA	0.911 (837)	1.464 (911)	-0.516 (355)	1.332 (889)
C020	Camorin-Jacarepaguá (Brazil)	22°56’ 43°22’	5	8.4	6.0	23.1	1229	-0.5	Tropical and Subtropical Moist Broadleaf Forests	ATL	-1.463* (22)	-1.679 (27)	-0.862 (163)	-1.458 (53)
C023	Campinas (Brazil)	22°50’ 47°08’	18	11.7	6.4	19.8	1302	-0.5	Tropical and Subtropical Moist Broadleaf Forests	ATL	-0.379 (404)	-0.506 (294)	-0.577 (280)	-0.151 (450)
C025	Río Candamo (Peru)	13°30’ 69°50’	3	9.9	3.2	24.2	3694	19.0	Tropical and Subtropical Moist Broadleaf Forests	AMA	-1.07 (199)	-1.2 (210)	-1.576* (15)	-1.122 (91)
C027	Capeira (Ecuador)	02°00’ 79°58’	5	10.0	2.5	25.4	738	-12.0	Tropical and Subtropical Moist Broadleaf Forests	DRY	0.848 (169)	1.404 (914)	-0.543 (339)	1.338 (890)
C028	Carajás (Brazil)	05°30’ 51°00’	12	11.0	1.0	25.3	1875	6.0	Tropical and Subtropical Moist Broadleaf Forests	AMA	-1.273 (43)	0.131 (546)	-0.986 (62)	0.458 (661)
C029	Carara National Park (Costa Rica)	09°46’ 84°32’	5	10.0	2.5	26.6	2902	8.5	Tropical and Subtropical Moist Broadleaf Forests	CEN	-0.364 (412)	-0.597 (296)	-1.026 (107)	-0.317 (388)
C030	São Carlos (Brazil)	21°57’ 47°50’	18	11.1	5.8	20.1	1398	-0.5	Tropical and Subtropical Grasslands, Savannas, and Shrublands	ATL	-0.422 (361)	-1.678 (48)	-0.219 (535)	-1.196 (95)
C033	Carlos Botelho (Brazil)	24°15’ 46°56’	3	8.1	7.2	22.6	2039	10.5	Tropical and Subtropical Moist Broadleaf Forests	ATL	-0.776 (209)	-1.01 (168)	-0.789 (235)	-0.979 (142)
C036	Cedral (Colombia)	04°45’ 75°33’	2	8.9	1.0	14.1	2240	16.0	Tropical and Subtropical Moist Broadleaf Forests	AMA	2.141 (933)	2.141 (933)	1.158 (847)	2.477 (947)

C037	Cerro de Neblina 1 (Venezuela)	00°50’ 66°11’	2	10.0	1.1	26.0	3152	23.0	Tropical and Subtropical Moist Broadleaf Forests	AMA	-0.451 (352)	-0.451 (352)	0.152 (597)	-0.559 (260)
C038	Cerro de Neblina 2 (Venezuela)	00°50’ 66°11’	3	10.0	1.1	26.0	3152	23.0	Tropical and Subtropical Moist Broadleaf Forests	AMA	-1.203* (12)	-1.344* (11)	-1.672* (5)	-1.281 (48)
C039	Cerro Oluma (Nicaragua)	12°18’ 85°24’	5	9.5	2.9	23.2	1542	4.5	Tropical and Subtropical Coniferous Forests	CEN	0.037 (573)	-0.162 (455)	0.379 (728)	0.048 (552)
C041	Chamela Arroyos (Mexico)	19°30’ 105°03’	5	12.8	4.4	26.4	743	-11.5	Tropical and Subtropical Dry Broadleaf Forests	DRY	-1.056 (115)	-0.654 (264)	-1.079 (89)	-0.516 (306)
C042	Chaquimayo (Bolivia)	14°34’ 68°28’	10	9.1	3.5	20.9	1572	3.5	Tropical and Subtropical Moist Broadleaf Forests	AMA	0.493 (733)	-0.276 (410)	-0.004 (628)	0.073 (551)
C048	Cocha Cashu (Peru)	11°51’ 71°19’	11	11.2	2.4	25.0	2490	14.5	Tropical and Subtropical Moist Broadleaf Forests	AMA	-0.512 (349)	-1.803 (42)	-0.906 (96)	-1.025 (136)
C050	Loma de los Colorados (Colombia)	09°58’ 75°10’	12	10.9	1.4	27.2	1563	4.5	Tropical and Subtropical Moist Broadleaf Forests	AMA	-0.749 (246)	0.339 (638)	-0.562 (297)	0.539 (702)
C051	Colosó 1 (Colombia)	09°30’ 75°48’	7	8.7	1.3	27.6	864	-2.0	Deserts and Xeric Shrublands	DXS	-0.416 (355)	-0.433 (276)	1.444 (917)	-0.334 (377)
C052	Constancia (Peru)	04°15’ 72°45’	3	9.6	1.6	26.1	2680	19.0	Tropical and Subtropical Moist Broadleaf Forests	AMA	-1.181 (79)	-1.286 (78)	-0.887 (193)	-1.307 (37)
C054	Cuangos (Ecuador)	03°29’ 78°14’	4	10.8	1.9	20.7	3066	22.0	Tropical and Subtropical Moist Broadleaf Forests	AMA	0.939 (844)	0.481 (717)	-0.291 (465)	0.605 (770)
C057	Curundu (Panama)	08°59’ 79°33’	5	7.9	1.8	27.0	1865	8.0	Tropical and Subtropical Moist Broadleaf Forests	CEN	0.469 (742)	1.035 (851)	-0.761 (232)	1.002 (837)
C058	Curuyuqui Riverine (Bolivia)	18°45’ 62°18’	2	13.0	6.7	24.8	578	-19.0	Tropical and Subtropical Dry Broadleaf Forests	DRY	-0.294 (432)	-0.294 (432)	2.348* (995)	-0.432 (387)
C060	Cuzco Amazónico (Peru)	12°35’ 69°09’	11	11.2	3.1	25.5	2183	9.5	Tropical and Subtropical Moist Broadleaf Forests	AMA	-0.337 (412)	-0.72 (229)	-0.052 (597)	-0.475 (330)
D062	Reserva Ducke (Brazil)	03°00’ 58°58’	5	8.4	1.4	27.3	2180	12.5	Tropical and Subtropical Moist Broadleaf Forests	AMA	1.37 (903)	1.628 (938)	-0.049 (592)	1.585 (913)
D063	Dureno (Ecuador)	00°15’ 76°45’	3	9.7	1.5	24.8	3450	24.0	Tropical and Subtropical Moist Broadleaf Forests	AMA	1.491 (918)	2.618* (990)	0.089 (619)	2.831* (993)
E065	El Encanto (Bolivia)	14°38’ 60°42’	15	13.8	4.0	21.9	1481	3.5	Tropical and Subtropical Dry Broadleaf Forests	AMA	0.625 (764)	-0.866 (190)	-0.44 (376)	-0.337 (358)
E066	Esmeraldas Tropical Garden (Ecuador)	00°54’ 79°37’	10	6.8	1.3	21.9	944	-9.5	Tropical and Subtropical Moist Broadleaf Forests	DRY	1.429 (921)	-1.009 (159)	0.035 (672)	-0.43 (323)
F067	Farallones de Cali (Colombia)	03°30’ 76°35’	2	11.0	1.1	19.9	1512	4.0	Tropical and Subtropical Moist Broadleaf Forests	AMA	0.806 (768)	0.806 (768)	-0.187 (470)	0.968 (798)
F068	Paulo de Faria (Brazil)	19°56’ 49°31’	14	13.0	4.9	24.0	1247	-1.5	Tropical and Subtropical Moist Broadleaf Forests	ATL	0.41 (708)	0.839 (787)	0.175 (743)	1.179 (881)
G070	Galerazamba (Colombia)	10°48’ 75°15’	3	7.4	4.9	27.7	864	-8.0	Deserts and Xeric Shrublands	DXS	0.408 (746)	0.2952 (659)	-0.417 (424)	0.358 (676)

G073	Guanacaste gallery	10°30’	4	10.4	2.4	26.5	1580	-0.5	Tropical and Subtropical Dry	DRY	-0.228	-0.377	0.306	-0.306
	forest (Costa Rica)	85°10’							Broadleaf Forests		(505)	(378)	(687)	(396)
G074	Guanacaste upland	10°32’	5	10.7	2.5	26.1	1632	0.5	Tropical and Subtropical Dry	DRY	1.853	1.756	0.032	1.665
	(Costa Rica)	85°18’							Broadleaf Forests		(950)	(955)	(591)	(923)
H077	Bosque Nacional	08°50’	11	11.7	1.5	26.1	2493	15.5	Tropical and Subtropical Moist	AMA	1.147	-0.324	-0.444	0.046
	von Humboldt (Peru)	75°00’							Broadleaf Forests		(887)	(357)	(363)	(534)
I080	Indiana (Peru)	03°31’	7	9.8	1.7	26.4	2804	22.0	Tropical and Subtropical Moist	AMA	-0.079	-0.797	-0.562	-0.407
		73°04’							Broadleaf Forests		(549)	(217)	(327)	(358)
J087	Jatun Sacha	01°04’	5	9.9	1.3	23.9	3654	23.0	Tropical and Subtropical Moist	AMA	0.042	0.276	-0.924	0.424
	(Ecuador)	77°36’							Broadleaf Forests		(598)	(635)	(152)	(707)
J088	Jauneche (Ecuador)	01°06’	12	9.1	2.2	25.4	1831	-2.0	Tropical and Subtropical Moist	DRY	2.367	0.496	0.223	0.661
		79°38’							Broadleaf Forests		(976)	(699)	(752)	(759)
J089	Río Jejuimi	24°08’	10	11.4	9.4	22.5	1612	6.5	Tropical and Subtropical Moist	ATL	-1.081	-1.386	-1.004	-0.732
	(Paraguay)	55°32’							Broadleaf Forests		(101)	(76)	(71)	(220)
J090	Jenaro Herrera	04°55’	5	10.1	1.4	26.9	2615	20.0	Tropical and Subtropical Moist	AMA	0.412	-0.869	-0.806	-0.602
	(Peru)	73°45’							Broadleaf Forests		(706)	(209)	(192)	(287)
L092	La Genoa (Peru)	11°05’	5	13.7	2.3	18.6	1578	7.5	Tropical and Subtropical Moist	AMA	-0.37	-0.452	-0.722	-0.279
		75°25’							Broadleaf Forests		(399)	(332)	(247)	(399)
L093	La Raya (Colombia)	08°20’	5	10.1	1.4	28.0	3083	8.5	Tropical and Subtropical Moist	AMA	-1.043	-0.226	-1.172	-0.007
		74°55’							Broadleaf Forests		(123)	(426)	(44)	(546)
L094	La Selva (Costa	10°26’	7	8.8	2.4	26.0	3974	22.0	Tropical and Subtropical Moist	CEN	0.011	-0.381	-1.032	-0.001
	Rica)	84°01’							Broadleaf Forests		(575)	(355)	(102)	(539)
L098	Linhares (Brazil)	19°18’	8	9.2	5.0	24.1	1207	-2.0	Tropical and Subtropical Moist	ATL	-0.486	-1.392	-1.118	-0.698
		40°04’							Broadleaf Forests		(350)	(73)	(53)	(224)
L099	Estación Biológica de	08°56’	4	10.5	3.0	27.4	1209	-2.5	Tropical and Subtropical Moist	SAV	1.701	1.459	-0.002	1.443
	los Lanos (Venezuela)	67°25’							Broadleaf Forests		(934)	(908)	(604)	(896)
M103	Madden Forest	09°06’	7	7.5	2.0	26.1	2185	8.0	Tropical and Subtropical Moist	CEN	0.222	-0.01	-0.85	0.204
	(Panama)	79°36’							Broadleaf Forests		(650)	(518)	(160)	(626)
M104	Alto Madidi	13°35’	15	10.3	3.4	25.1	2293	11.5	Tropical and Subtropical Moist	AMA	0.484	-1.399	-0.845	-0.541
	(Bolivia)	68°46’							Broadleaf Forests		(711)	(73)	(112)	(284)
M105	Alto Madidi, ridge	13°35’	7	10.3	3.4	25.1	2293	11.5	Tropical and Subtropical Moist	AMA	0.633	-0.473	-0.192	-0.155
	top (Bolivia)	68°46’							Broadleaf Forests		(797)	(332)	(525)	(461)
M106	Magsasay (Costa	10°24’	8	8.9	2.4	25.5	4132	23.0	Tropical and Subtropical Moist	CEN	-0.068	-0.727	-0.831	-0.366
	Rica)	84°03’							Broadleaf Forests		(532)	(244)	(161)	(366)
M107	Manaure	10°22’	3	11.7	1.6	27.2	1287	-0.5	Tropical and Subtropical Moist	AMA	-0.172	-0.415	-0.552	-0.461
	(Colombia)	73°08’							Broadleaf Forests		(479)	(399)	(333)	(415)
M108	Manaus (Brazil)	03°08’	5	8.3	1.3	27.5	2148	12.5	Tropical and Subtropical Moist	AMA	-0.885	-0.291	-1.074	-0.058
		60°01’							Broadleaf Forests		(157)	(408)	(92)	(515)
M110	Mariquita	05°15’	3	10.0	1.3	26.7	2020	13.0	Tropical and Subtropical Dry	DRY	-0.609	-0.939	-0.622	-0.963
	(Colombia)	74°50’							Broadleaf Forests		(309)	(232)	(324)	(183)

M111	Miazi (Ecuador)	04°18' 78°40'	5	11.3	2.1	22.0	2270	17.0	Tropical and Subtropical Moist Broadleaf Forests	AMA	-1.439* (8)	-1.656* (12)	2.373 (965)	-2.254* (4)
M112	Mishana Old Floodplain (Peru)	03°47' 73°30'	8	9.8	1.6	26.3	2845	22.0	Tropical and Subtropical Moist Broadleaf Forests	AMA	0.185 (614)	-0.413 (349)	-0.682 (248)	-0.105 (481)
M113	Mishana White Sand (Peru)	03°47' 73°30'	4	9.8	1.6	26.3	2845	22.0	Tropical and Subtropical Moist Broadleaf Forests	AMA	-1.156 (59)	-1.31 (78)	-0.548 (343)	-1.284 (83)
M118	Río Nangaritza (Ecuador)	04°18' 78°40'	4	11.3	2.1	22.0	2270	17.0	Tropical and Subtropical Moist Broadleaf Forests	AMA	-0.169 (493)	-0.257 (316)	0.315 (740)	-0.315 (547)
N119	Nuevo Mundo (Bolivia)	10°39' 66°46'	10	11.0	2.3	26.2	1783	4.5	Tropical and Subtropical Moist Broadleaf Forests	AMA	1.83 (955)	1.169 (866)	0.165 (712)	1.281 (885)
O121	Orleans (Brazil)	28°21'32" 49°17'29"	4	10.5	8.7	19.4	1450	6.5	Tropical and Subtropical Moist Broadleaf Forests	ATL	-0.181 (496)	0.108 (564)	-0.362 (438)	0.288 (670)
P124	São Paulo (Brazil)	23°33' 46°43'	10	9.9	6.3	18.5	1403	0.5	Tropical and Subtropical Moist Broadleaf Forests	ATL	-0.351 (424)	-1.62 (51)	-0.749 (185)	-0.872 (183)
P126	Perro Muerto (Ecuador)	01°36' 80°42'	6	8.1	2.3	22.3	567	-15.5	Tropical and Subtropical Dry Broadleaf Forests	DRY	0.461 (724)	-0.881 (201)	-0.026 (601)	-0.624 (278)
P127	Perseverancia (Bolivia)	14°38' 62°37'	17	12.7	4.1	24.9	1292	-4.5	Tropical and Subtropical Moist Broadleaf Forests	AMA	-0.15 (500)	-0.021 (473)	-0.732 (168)	0.321 (663)
P128	Pipeline Road (Panama)	09°10' 79°45'	4	7.0	2.4	25.7	2598	9.5	Tropical and Subtropical Moist Broadleaf Forests	CEN	-0.372 (402)	-0.602 (290)	-0.81 (224)	-0.432 (367)
Q131	Quiapaca (Bolivia)	18°20' 59°30'	13	12.9	5.8	25.2	1114	-3.5	Tropical and Subtropical Dry Broadleaf Forests	DRY	0.623 (773)	0.239 (595)	-0.083 (594)	0.551 (717)
R132	Rancho Quebrado (Costa Rica)	08°42' 83°33'	2	11.2	2.4	25.3	3861	13.5	Tropical and Subtropical Moist Broadleaf Forests	CEN	-0.339 (407)	-0.337 (407)	-0.671 (327)	-0.397 (416)
R133	Arroyo Riachuelo (Argentina)	27°00' 58°00'	2	10.8	10.9	22.1	1146	-0.5	Tropical and Subtropical Grasslands, Savannas, and Shrublands	SAV	-0.277 (447)	-0.277 (447)	2.23* (983)	-0.387 (396)
R134	Pampas del Heath (Peru)	12°50' 68°50'	10	11.0	3.3	25.5	2142	9.5	Tropical and Subtropical Moist Broadleaf Forests	AMA	0.64 (776)	-0.855 (186)	-0.502 (345)	-0.341 (392)
R135	Río Manso (Colombia)	07°30' 76°05'	7	9.9	1.3	25.6	2964	14.5	Tropical and Subtropical Moist Broadleaf Forests	AMA	-1.262 (42)	-1.014 (149)	-0.952 (115)	-0.69 (250)
R136	Río Negro (Bolivia)	9°50' 65°40'	9	10.7	2.0	26.4	1609	1.5	Tropical and Subtropical Moist Broadleaf Forests	AMA	0.399 (693)	-0.344 (374)	-0.655 (251)	0.08 (554)
R137	Río Palenque 1 (Ecuador)	00°34' 79°20'	2	8.7	2.4	23.8	2702	5.0	Tropical and Subtropical Moist Broadleaf Forests	AMA	2.693 (968)	2.693 (968)	1.614 (922)	3.123 (974)
R138	Río Palenque 2 (Ecuador)	00°34' 79°20'	3	8.7	2.4	23.8	2702	5.0	Tropical and Subtropical Moist Broadleaf Forests	AMA	2.399 (974)	2.165 (968)	1.13 (847)	2.44 (960)
R139	Río Távara (Peru)	13°21' 69°40'	11	10.1	3.3	24.2	3509	19.0	Tropical and Subtropical Moist Broadleaf Forests	AMA	-0.301 (438)	-0.207 (427)	-0.907 (97)	0.156 (582)
S142	San Sebastián (Ecuador)	01°36' 80°42'	2	8.1	2.3	22.3	567	-16.0	Tropical and Subtropical Dry Broadleaf Forests	DRY	0.749 (755)	0.749 (755)	-0.221 (455)	1.041 (817)

S143	Santa Cruz (Bolivia)	17°46’ 63°04’	12	10.8	6.1	24.4	1080	-6.5	Tropical and Subtropical Dry Broadleaf Forests	DRY	-1.523* (19)	-1.658 (56)	-0.78 (168)	-1.128 (117)
S145	Lomas de Santo Tomás (Colombia)	04°55’ 74°50’	6	10.7	1.1	27.4	1465	0.5	Tropical and Subtropical Dry Broadleaf Forests	DRY	-0.561 (336)	-0.436 (341)	-0.876 (160)	-0.15 (450)
S146	Saul (French Guiana)	03°38’ 53°12’	5	9.0	1.5	23.9	2506	15.5	Tropical and Subtropical Moist Broadleaf Forests	AMA	-0.163 (510)	-0.295 (405)	-0.854 (175)	-0.087 (486)
S147	Shiringamazú (Peru)	10°20’ 75°10’	5	11.7	1.4	24.0	2651	17.0	Tropical and Subtropical Moist Broadleaf Forests	AMA	-0.15 (518)	-0.136 (466)	-0.654 (292)	0.024 (539)
S151	Quebrada Sucusari (Peru)	03°15’ 72°55’	4	9.7	1.6	26.4	2813	22.0	Tropical and Subtropical Moist Broadleaf Forests	AMA	0.166 (604)	1.849 (950)	-0.501 (365)	1.794 (927)
T152	Mishana Tahuampa (Peru)	03°47’ 73°30’	4	9.8	1.6	26.3	2845	22.0	Tropical and Subtropical Moist Broadleaf Forests	AMA	-0.845 (195)	-0.703 (237)	-0.402 (427)	-0.603 (295)
T153	Tambopata Swamp Trail (Peru)	12°47’ 69°17’	10	11.0	3.3	25.4	2424	11.5	Tropical and Subtropical Moist Broadleaf Forests	AMA	-0.092 (518)	0.391 (652)	-0.412 (399)	0.588 (723)
T154	Tambopata Lateritic (Peru)	12°47’ 69°17’	13	11.0	3.3	25.4	2424	11.5	Tropical and Subtropical Moist Broadleaf Forests	AMA	-0.901 (139)	-0.885 (191)	-1.095* (19)	-0.344 (357)
T155	Tambopata Alluvial (Peru)	12°50’ 69°17’	9	11.0	3.3	25.4	2458	12.5	Tropical and Subtropical Moist Broadleaf Forests	AMA	-1.114 (77)	-2.393* (6)	-1.082 (29)	-1.508 (62)
T156	Tambopata Upland Sandy (Peru)	12°49’ 69°43’	12	10.6	3.4	25.0	3095	19.0	Tropical and Subtropical Moist Broadleaf Forests	AMA	0.577 (750)	-0.06 (472)	-0.378 (409)	0.187 (603)
T157	Tarapoto (Peru)	06°35’ 76°25’	6	13.0	1.4	25.2	1125	-3.0	Tropical and Subtropical Moist Broadleaf Forests	AMA	-0.255 (479)	0.497 (709)	0.143 (654)	0.699 (786)
T158	Tayrona National Park (Colombia)	11°20’ 74°02’	6	9.9	1.8	25.9	1603	2.5	Deserts and Xeric Shrublands	DRY	0.463 (703)	0.487 (687)	-0.546 (331)	0.656 (766)
T161	Los Tuxtlas (Mexico)	18°35’ 95°08’	4	8.5	5.6	20.4	2834	11.5	Tropical and Subtropical Moist Broadleaf Forests	CEN	-0.062 (541)	-0.4191 (381)	-0.842 (191)	-0.285 (413)
U163	Boca de Uchire (Venezuela)	10°08’48” 65°25’40”	9	10.1	2.1	27.5	653	-16.0	Deserts and Xeric Shrublands	DXS	0.917 (845)	0.709 (755)	0.317 (742)	1.014 (837)
V164	Viamão (Brazil)	30°05’ 50°51’	5	9.4	10.2	19.1	1470	10.5	Tropical and Subtropical Grasslands, Savannas, and Shrublands	ATL	-0.0217 (529)	-0.583 (306)	-0.558 (326)	-0.323 (400)
Y166	Yanamono 1 (Peru)	03°26’ 72°51’	8	9.6	1.7	26.2	2809	21.0	Tropical and Subtropical Moist Broadleaf Forests	AMA	-1.146 (73)	-0.481 (327)	-1.397* (1)	-0.058 (523)
Y167	Yanamono 2 (Peru)	03°26’ 72°51’	5	9.6	1.7	26.2	2809	21.0	Tropical and Subtropical Moist Broadleaf Forests	AMA	-0.191 (497)	0.827 (780)	-1.218 (58)	0.836 (808)
Y168	Yanamono Tahuampa (Peru)	03°28’ 72°50’	7	9.6	1.6	26.3	2833	21.0	Tropical and Subtropical Moist Broadleaf Forests	AMA	-0.495 (351)	-1.507 (57)	-1.029 (106)	-0.966 (163)
